# Automatic Assessment of Procedural Skills Based on the Surgical Workflow Analysis Derived from Speech and Video †

**DOI:** 10.3390/bioengineering9120753

**Published:** 2022-12-02

**Authors:** Carmen Guzmán-García, Patricia Sánchez-González, Ignacio Oropesa, Enrique J. Gómez

**Affiliations:** 1 Biomedical Engineering and Telemedicine Centre, ETSI Telecomunicación, Center for Biomedical Technology, Universidad Politécnica de Madrid, 28040 Madrid, Spain; 2 Centro de Investigación Biomédica en Red en Bioingeniería, Biomateriales y Nanomedicina, 28029 Madrid, Spain

**Keywords:** procedural skills, surgical training, skills’ assessment, artificial intelligence

## Abstract

Automatic surgical workflow analysis (SWA) plays an important role in the modelling of surgical processes. Current automatic approaches for SWA use videos (with accuracies varying from 0.8 and 0.9), but they do not incorporate speech (inherently linked to the ongoing cognitive process). The approach followed in this study uses both video and speech to classify the phases of laparoscopic cholecystectomy, based on neural networks and machine learning. The automatic application implemented in this study uses this information to calculate the total time spent in surgery, the time spent in each phase, the number of occurrences, the minimal, maximal and average time whenever there is more than one occurrence, the timeline of the surgery and the transition probability between phases. This information can be used as an assessment method for surgical procedural skills.

## 1. Introduction

Minimally invasive surgery (MIS) has evolved as the gold standard for highly accurate, sensitive and less invasive surgical procedures [1]. These procedures have gained clinical acceptance because of their advantages compared to open surgery, such as less pain, less scarring, less damage to healthy tissue and a faster recovery [1]. However, the rapidly progressing initial implementations of MIS techniques has led to an alarming number of complications because of inadequately trained and skilled surgeons [2]. As a result, surgical education shifted to provide learners with the necessary MIS skills, so that the effectiveness and efficiency of surgical training could be optimised without patients being at risk [3].

Advanced performance in MIS depends on the combination of various skill sets [4,5], for which any objective training and assessment must be tailor-made [6] and rigorously validated [7]. These skills range from technical (e.g., dexterity, depth perception, efficiency, autonomy, etc.) to nontechnical (e.g., stress management, decision-making, etc.), the latter including advanced cognitive skills (e.g., conceptual and procedural knowledge) [3]. 

Residents and educators alike have been particularly concerned about advanced cognitive skills’ acquisition and have been looking at strategies to combat this lack of experience [8]. Advanced cognitive skills are defined as the “core principles that guide the thoughts, judgements, decisions, and actions during surgical performance” [4]. 

One of the most extended strategies for the training and assessment of advanced cognitive skills (and specifically procedural skills) is the analysis of surgical processes targeted at the automatic recognition of surgical phases. 

Surgical processes are defined as “a set of one or more linked procedures or activities that collectively realise a surgical objective within the context of an organisational structure” [9]. The key aspect of the analysis of surgical processing is surgical process modelling (SPM), which has been defined as a “simplified pattern of surgical processes that reflect a predefined subset of interest of the surgical process in a formal or semi-formal representation” [9].

Surgical processes can be modelled at any level (i.e., modelling the whole procedure or modelling a specific task within it). These levels are known as granularity levels. A granularity level is defined as the level of abstraction at which the surgical procedure is described [10]. MacKenzie et al. [11] proposed a hierarchical model of the surgical procedure that consists of different levels of granularity.

The highest level is the procedure itself, which is composed by a list of phases (defined as the major types of events occurring during surgery). Each phase is composed of several steps or tasks (considered to be a sequence of activities used to achieve a surgical objective). An activity is a physical task and is composed by a list of motions (considered to be a surgical task involving only one hand trajectory with no meaning by itself, in the sense that it can be a task performed in any arbitrary procedure, and only acquire meaning when linked to a specific activity). Each granularity level is assumed to describe the surgical procedure as a sequential list of events, except for the surgical procedure itself and lower levels of granularity (e.g., image, video, presence, etc.) where information may be continuous [10].

For instance, Uemura et al. [3] employed an SPM software to manually tag each task performed during the surgery. Then, they extracted metrics (e.g., number of ocurrences, average duration, occupancy percentage…) to develop an assessment method for procedural and decision-making skills necessary when facing complications in the OR. 

On the other hand, Loukas et al. [12] used a different annotation tool to analyze the order of surgical tasks through performance metrics (i.e., surgeme counts, duration of each surgeme, counts of penalty-events and counts of surgeme transitions). They were able to recognize trainees’ skill level with 0.71 accuracy.

SPM was first introduced for supporting whole surgical interventions, but soon their applications in surgical training gained popularity amongst the surgical educational community [10]. Firstly, it could be used to constitute a universal model of surgical procedures which could unify their training in all medical institutions. Secondly, it could facilitate the training and assessment of advanced cognitive skills for said procedures. All of this has a direct impact on patient safety, since patient’s positioning could be anticipated, operating time optimised or technical requirements for the procedure analysed before the intervention [10]. In addition to this, the model of the surgical process could be useful for designers of procedural surgical simulators, replicating not only the process itself but also potential complications and solutions to overcome them.

One of the steps involved in surgical process modelling (SPM) is surgical workflow analysis (SWA), which is the deconstructing process by which a surgical process is divided into a list of different phases, activities or tasks [13].

Currently, SWA focuses on analysing the laparoscopic video to recognize surgical phases and activities using artificial intelligence. Probabilistic methods have proven to be of great interest in video-based SWA [14,15]. However, they are usually trained with simple features based on low-level visual cues, typically obtained through a manual annotation process. This manual (human-based) annotation is virtually impossible to perform for real-time applications [16]. Thus, recent video-based SWA studies have shifted towards the recognition of surgical phases using a combination of deep learning techniques and probabilistic methods (mostly with Hidden Markov Models –HMMs–) [16,17,18,19]. The best results obtain accuracies varying from 0.8 to 0.9, approximately [16,18,20,21].

A limitation of video-based analysis is that it is based on outcomes (the surgical action) rather than on the mental process that triggers it. Thus, our current line of research considers monitoring audio cues during surgical procedures from the attending surgical staff, motivated by the Think Aloud theory by which verbalization of tasks are inherently linked to the ongoing cognitive processes [22]. To our knowledge, the only study in this field was reported by Suzuki et al., which attempted to use audio information to find patterns of conversation (i.e., correlating the amount of conversation to the probability of transitioning from one phase of the procedure to another) [23]. However, their recognition algorithm reached only a 51.6% accuracy. 

We explored this avenue in a previous study [24], where we used the surgeon-in-training’s speech to classify surgical phases using natural language processing (NLP) and machine learning, reaching a 80.7% accuracy. In order to increase the robustness of the algorithm, our current research is focused on combining visual information and speech during a surgical procedure [25]. 

The main goal of this study is to implement an intelligent system to train and assess surgeons’ procedural skills by means of SWA. The system will integrate a surgical phase classifier (i.e., an algorithm capable of classifying surgical phases combining visual information and speech from a surgical procedure). To do this, we propose to create a desktop application to open videos and obtain and display metrics using the results from the surgical phase classifier. This system could also serve as a training tool to guide surgical residents in the procedure.

In this study, we present both the application for procedural skills’ assessment and the algorithm for surgical phase classification based on speech and video, including preliminary results obtained from a test video.

## 2. Materials and Methods

### 2.1. Data

Laparoscopic cholecystectomy (LC) was selected as the main procedure for both studies to analyze since it is a widespread and convenient procedure for SWA (due to the unique characteristics of each of its phases), as well as one of the interventions with the highest training rate [16,26]. 

**Figure 1:**
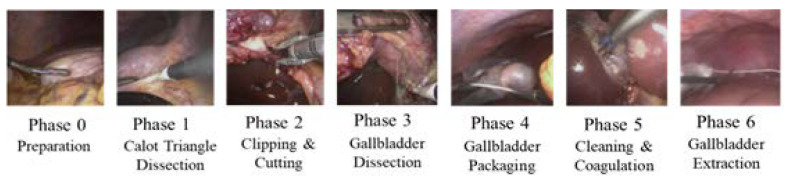
Phases of LC.

To train the endoscopic video model, the dataset from the “Surgical Workflow and Skill Analysis” sub-challenge of the Endoscopic Vision Challenge [27] was used. This dataset consists of 24 endoscopic videos obtained during LC procedures at the University Hospital of Heidelberg and its affiliate hospitals. Surgical phases for all interventions were annotated frame-wise by at least two surgical experts. The phases of the procedure are defined in Figure 1.

Additionally, to account for those audio signals including sentences that are not related to any of these phases (i.e., normal conversation occurring in the OR, questions posed by teachers and/or students, etc.), we included a pseudo-phase in the audio-based model.

To train the speech model, a database was created using 15 surgical educational videos found online and taken in different hospitals, in which the steps of the LC were verbally disclosed in Spanish by a single trainer surgeon equipped with a clip-on microphone [24]. The audio from these videos was extracted and fragmented into smaller pieces according to the silences in the recording, using Python’s speech_recognition module [28]. This allowed for having smaller audio sequences which could be easily associated with a specific LC phase or with the pseudo-phase.

To evaluate the performance of the application, two educational videos were used. These videos, similar to the ones in which the speech dataset is based, consisted of high-quality endoscopic video feeds found online on top of which the surgeon verbally discloses the ongoing procedure. One of them contains a total of 51 audio samples and 367 video samples (frames), whereas the other contains 28 audio samples and 269 frames. All samples were then manually tagged independently following the same guidelines as for the individual datasets.

### 2.2. Algorithm for Surgical Phase Classification Based on Speech and Video

The implemented algorithm for the recognition of surgical phases uses two different classification models: one for the endoscopic video feed, and one for the surgeon-in-training’s voice (obtained through of a clip-on microphone). 

**Figure 2:**
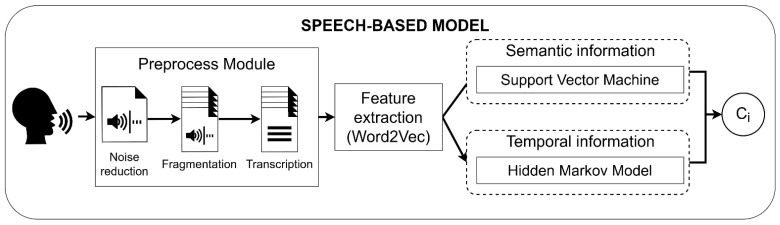
Workflow of the speech-based model. C_i_ represents the predicted class.

The audio and image channels are separated using FFmpeg (FFmpeg, Sofia, Bucharest). The speech signals are preprocessed to be divided into fragments and automatically transcribed into text using Google transcriber (Figure 2). Next, text data is transformed into feature vectors using Word2Vec. Word2Vec is a neural network which predicts the context of the word, and returns a feature vector representing said context [29,30]. Finally, the obtained features are passed through a model which combines the semantic information obtained from Support Vector Machines (SVM) with the temporal information obtained from Hidden Markov Models (HMM). In a previous study, the model obtained an average accuracy of 80.7%. Further information on the architecture and training approach of the speech-based model can be found in [24]. 

**Figure 3:**
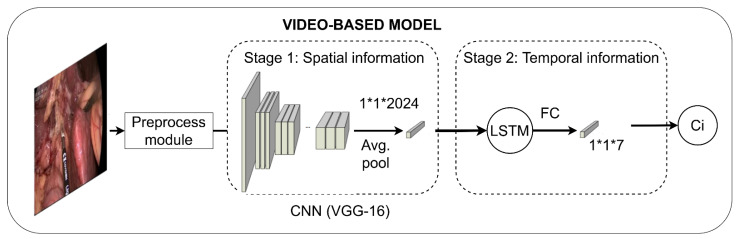
Workflow of the video-based model. FC represents a fully-connected layer; C_i_, the predicted class.

The endoscopic video is classified by means of a Convolutional Neural Network (CNN) with a Long Short-Term Memory (LSTM) unit (Figure 3). Specifically, the CNN is trained using a two-stage approach based on VGG-16 pretrained on ImageNet [31]. The hyperparameters were selected through a 21-fold cross-validation study in which a whole video from the training subset was left out for validation in each fold.

The first training stage learns the spatial information using transfer learning methodologies. This first stage takes frames as input and outputs the predicted class. The model resulting from this stage is used as a feature extractor, followed by an LSTM unit, such that the temporal information is learnt. LSTM units require sequences instead of individual frames to properly analyze the temporal dependencies. To accomplish this, sequences of nine frames in length, including the frame of interest and their four previous and four following frames, were extracted. The features from the individual frames included in these sequences were extracted from the model resulting from the first training stage and used to train the LSTM unit. 

Predictions from speech- and video-based models are synchronized in time. Specifically, the video samples are continuously processed until there is an audio sample obtained at the same moment. At this point, the predictions from both models are averaged until the audio signal ends. For instance, if an audio fragment took place between seconds 1–5, the phase predictions of the frames corresponding to these seconds were combined with the phase predictions of said audio fragment. 

Predictions from both audio and video models are given equal weight (i.e., 50% audio, 50% video). Thus, whenever there is more than one frame for a single audio fragment, the weight for the frames is equally divided to add up to 50%, and the other 50% is reserved for the audio fragment. These weights were selected to ensure the prediction power was the highest possible.

In addition, whenever the predictions of the audio-based model correspond to the pseudo-phase used to contain sentences not related to the procedure itself, they are eliminated from the analysis. Lastly, if no speech signal is captured (i.e., the surgeon is not speaking), only the video prediction is used as the final prediction.

### 2.3. Application for the Assessment of Advanced Cognitive Skills

**Figure 4:**
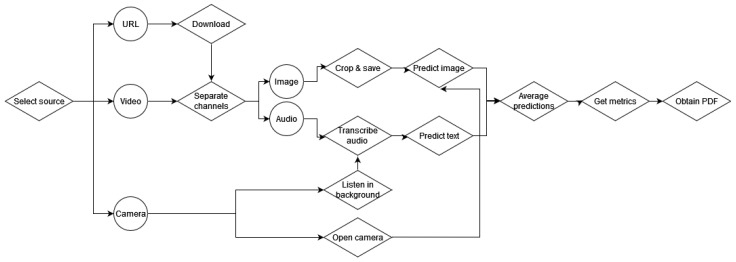
Flowchart of the application for the assessment of advanced cognitive skills.

The implemented intelligent system consists of a desktop application capable of obtaining audiovisual information, processing it accordingly, predicting the corresponding phase of the procedure, and obtaining a set of metrics related to procedural skills. Figure 4 depicts the flowchart of the application.

The application was implemented using Python and tkinter. It first allows the user to select the source from which to obtain the assessment metrics. The source can be the URL of an online video, a video in the PC or a webcam connected to the PC to perform real-time predictions. The video and URL options are included in case recorded interventions want to be assessed retroactively using this application.

**Figure 5:**
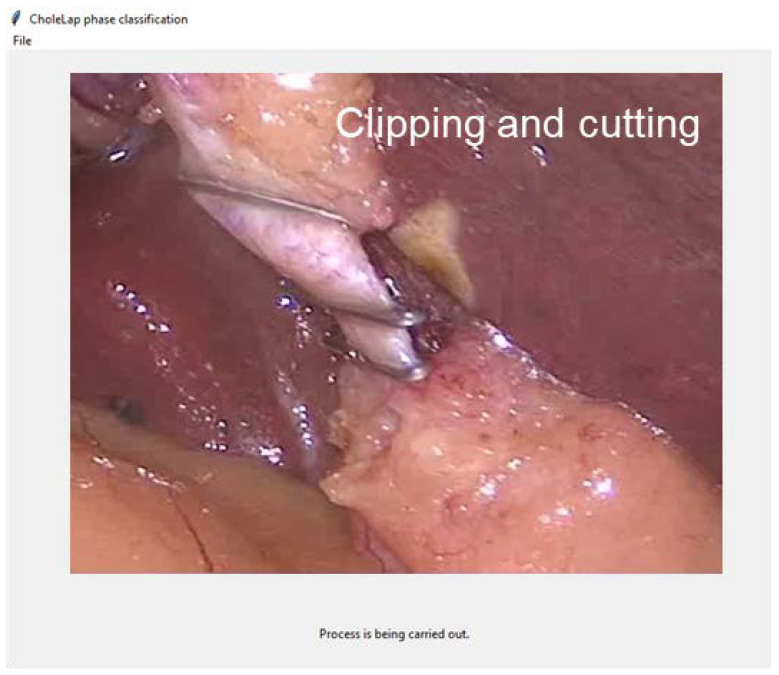
Screenshot of the application in real time while carrying out the prediction process.

If the URL is selected, the video will be downloaded and processed in the same manner as a local video. The local video (or the downloaded video from the URL) is read and the channels (audio and image) are separated. Then, images are cropped and saved locally to be processed by the image model, while audio is transcribed into text to be processed by the speech model. Images and text samples are classified according to their corresponding phase and averaged to obtain the final prediction. This prediction is printed on the screen together with the image corresponding to the video frame (Figure 5). 

In the case of real time predictions, the camera is opened, and images are fed to the video classification model, while the application uses the microphone to capture live conversation in the operating room (OR).

Once the video or streaming is over, the metrics are calculated. Specifically, the total time spent in surgery, the time spent in each phase, the number of occurrence of phases and the average time whenever there is more than one occurrence are calculated in an attempt to characterize the surgical process in a timewise way. These metrics have already been validated for the assessment of procedural skills in the study by Uemura et al. [3]. Metrics are coded into a timeline of phases where the *x*-axis represents the time in seconds and the colours correspond to the different phases. This allows for seeing the amount of time spent in each phase, the number of occurrences of phases and the percentage of time spent in each phase.

Lastly, to evaluate the assessment capability of the application, we have modeled an average workflow of the process using the samples in the video database [16], since it has been validated by clinicians. Specifically, the workflow was modeled based on the probabilities of transitioning from one phase to another, without taking into account the probability of remaining in a phase (i.e., the probability of going from a phase to the same phase). This is especially relevant in the video case, where consecutive frames are most likely to belong to the same phase. The probabilities of transitioning were obtained through the calculation of the transition probability matrix of the Markov chain generated by the workflow. The probabilities of transitioning from one phase to another were also calculated for the video or streaming of interest. The Wesserstein distance [32] between the probability distributions of the average workflow and the student’s workflow is calculated as a measure of the difference between the distributions.

All metrics and the workflow of phases are processed and printed in a PDF file. This can be used as a report of the performance of the student, both for the student to reflect on and for the teacher to follow their progress.

### 2.4. Evaluation

In order to assess the algorithm for phase recognition, performance was analyzed based on the F1 score, which is the harmonic mean of precision and recall. The F1 score of the complete algorithm was compared with that obtained by the video-based model and the speech-based model alone.

We analysed the processing time of different modules of the application (including the algorithm), which is essential for real-time feedback, by carrying out the process in the test video 10 times and averaging the processing times. Additionally, as a proof of concept, the overall workflow of the procedure (as obtained from the video database [16]) was compared with the workflow of the procedure followed by the surgeon-in-training. This will illustrate how the workflow can be of assistance for the assessment of procedural skills.

## 3. Results and discussion

### 3.1. Algorithm for Surgical Phase Classification Based on Audio and Video

Table 1 represents the F1 scores and errors obtained for the test video when using the video-based model, the speech-based model and the complete algorithm.

The combination of audio and video models resulted in a higher F1-score than for both individually (0.875). In addition, the average error per phase was found to be 11.86% and the average error, 17.89%, improving with respect to the individual models and the state of the art. This suggests that the algorithm we created to combine the two models was effective in its purpose and yields a more accurate prediction of surgical phases. This can be indicative of the ability of visual information to reinforce speech predictions (and vice versa), reducing the possibility of errors introduced by one of the models alone.

With respect to the video analysis alone, in the challenge in which the database we use was presented (HeiChole-19) [33], the resulting F1-scores ranged from 0.239 to 0.688, whereas in 2021 (HeiSurf-21), F1-scores varied from 0.529 to 0.703 [34]. The main results of these challenges can be found in Table 2. 

As seen in Table 2, two of the top performing methods use an encoder–decoder pretained on Cholec or ImageNet, reaching an F1-score of 0.703 and 0.661, respectively. The combination of CNN and LSTM seems to be a pattern for this specific problem, being used in six different algorithms. However, none of the methods reach an F1-score higher than 0.75, as is our case. 

**Figure 6:**
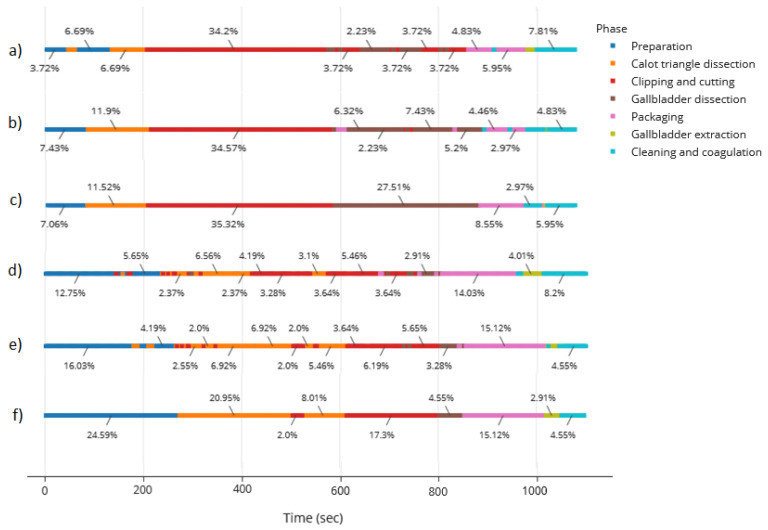
Timeline of phases for the videos in the test subset corresponding to (**a**) results of the first video using the video model alone, (**b**) results of the first video using the combined model, (**c**) ground truth for first video, (**d**) second video using the video model alone, (**e**) second video using the combined model, (**f**) ground truth for second video.

Figure 6 represents the timeline of phases for the videos in the test subset. It can be seen that, by combining the speech and video signal, the model achieves smoother results in both videos of the test subset as compared with the endoscopic video alone. However, in the second video, there are still some noisy patterns when going from the preparation to the Calot triangle dissection, confusing the latter with the clipping and cutting of the cystic artery and conduct. Despite the similarity between the field of view of these phases, we acknowledge a difference in the instrument used. In the future, we aim to create a model capable of distinguishing instruments to keep improving this combined model.

### 3.2. Application for the Assessment of Advanced Cognitive Skills

The time processing analysis resulted in an average 0.007 s to calculate the audio prediction (per speech sample), 0.057 s to calculate the video prediction (per frame) and 0.094 s to calculate the average predictions (per speech sample and frame). The PDF is generated within an average 2.164 s.

The time analysis shows that the video samples take more time to be classified than the speech samples. This may be due to the preprocessing associated to the video sample, which is necessarily longer than in the video case (i.e., predicting the features and creating the sequences). Nevertheless, the total time can be considered low for real time predictions, even more so taking into account that the variations between frames are usually small. 

In addition, the PDF greatly increases the processing time when embedding the plots into the document, although it does not affect the actual prediction and processing time. This could be solved by optimizing the plot creation, especially in the case of the workflow, which uses graphs to connect one phase to another. Moreover, the desktop application could be converted to a web application in which an HTML displays the graphs and the user decides whether or not to save it as a PDF.

The timeline of phases is especially useful to check whether the procedure was successfully followed. For instance, in the case studied in this work (Figure 6c), we observe that the phases were followed consecutively, except for the packaging phase, where extra cleaning and coagulation was necessary, forcing the surgeon to stop packaging to clean, finish the packaging and clean again to avoid post-surgical complications. The timeline provides a sense of the most time-consuming processes. This could be used, for example, to suggest to the surgeon-in-training the cut off time from a specific phase that could be considered too long.

**Figure 7:**
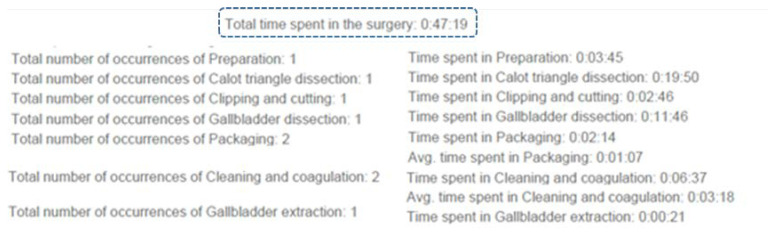
Example of the time and occurrences’ analysis.

The time and occurrences’ analysis allows for having a better idea of the time spent in each occurrence of phases. The time is represented as hours, minutes and seconds (Figure 7). This completes the information in the previous graph.

**Figure 8:**
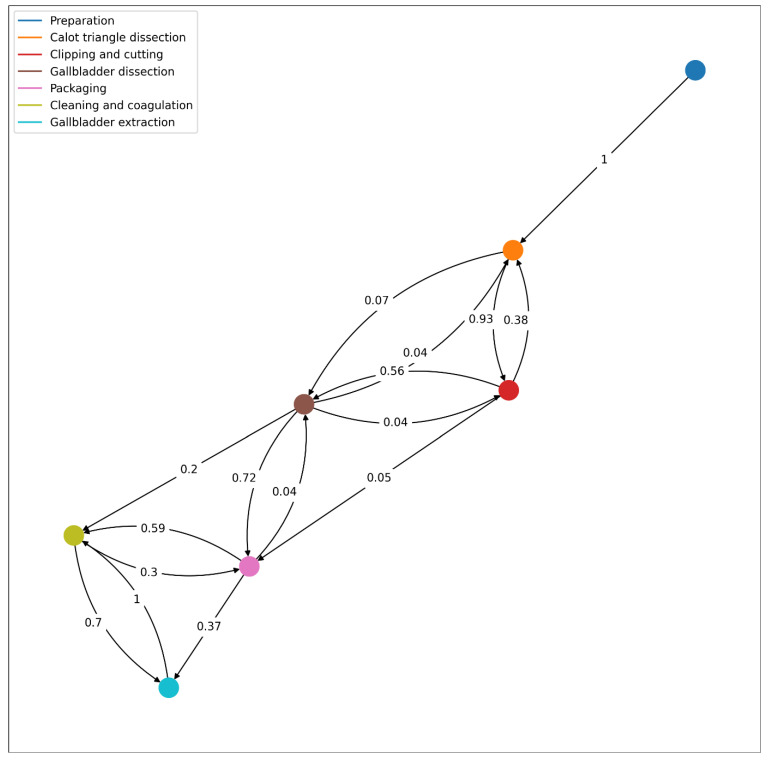
Workflow of the procedure based on the database from the “Surgical Workflow and Skill Analysis” sub-challenge of the Endoscopic Vision Challenge [20], with the corresponding transition probabilities from one phase to another.

Figure 8 introduces the workflow of the laparoscopic cholecystectomy obtained according to the video database [16]. 

**Figure 9:**
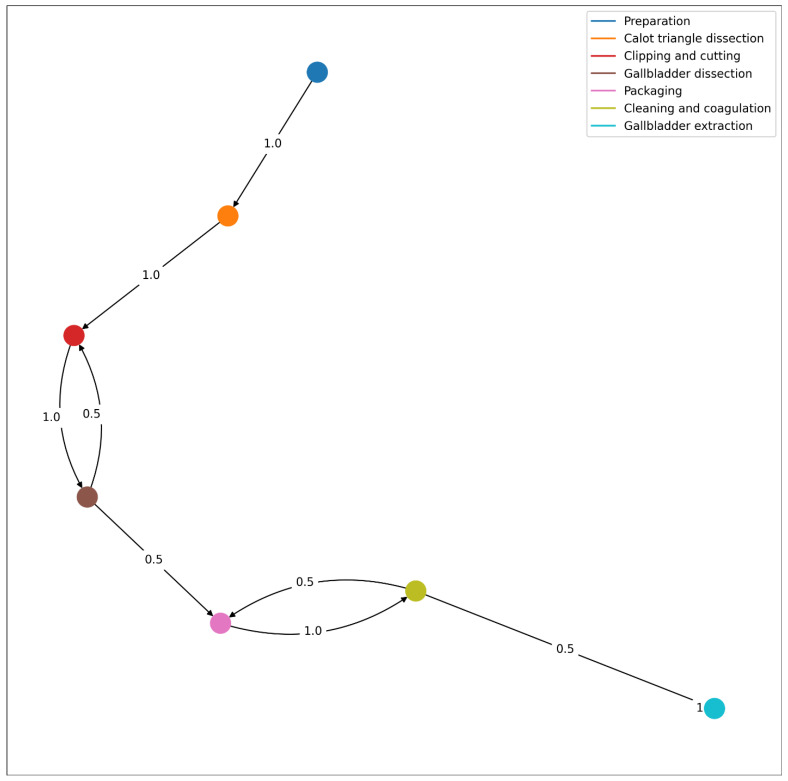
Workflow of the procedure described in the test video, with the corresponding transition probabilities from one phase to another.

Figure 9 represents the workflow followed by the surgeon of the test video for the integrated system. It is amongst the possible workflows as defined in the previously disclosed one. Analyzing the Wesserstein distance, we obtain that the highest differences are present in phases 3 (0.125) and 5 (0.116). Overall, the average Wesserstein distance is 0.078, which suggest that the students’ workflow is well within the model’s possibilities.

According to the resulting workflow from the integrated system, the surgeon in training successively goes from preparation, Calot’s triangle dissection, cystic duct and artery clipping and cutting and gallbladder dissection. This dissection was interrupted by the clipping (possibly because improved visibility was necessary). However, we know from the actual footage this was not the case and in fact, it is a misclassification of the integrated system. This stresses the need to improve the performance of the integrated system. The gallbladder dissection was followed by the gallbladder packaging, which in turn was followed by cleaning and coagulating. However, as pointed out before, while packaging, it may have been necessary to clean the anatomical surface and then continue packaging, which is why the surgeon in the test video went back to packaging from cleaning. The last phase is the gallbladder extraction (directly after cleaning and coagulating).

### 3.3. Limitations and Future Work

The main limitation of this study corresponds to the high computing times that it currently conveys. This makes the possibility for real-time applications (both for training and assessment) difficult. Thus, the first goal short-term is to optimize the algorithms to ensure the processing time is shortened and real-time applications are indeed possible. This can be done by introducing more threads to the application while respecting the main thread of the front-end.

On the other hand, although the model yields fairly accurate results, it fails to recognize phases, especially when first transitioning from one phase to another or whenever the field of view is too similar (e.g., dissection and clipping). In these cases, we aim at incorporating a model capable of recognizing instruments to incorporate additional information to help the model differentiate accurately between phases. We also plan to study a lower granularity of the procedure. Specifically, we will analyze the main actions in LC and annotate the speech samples accordingly. These new annotations will be used to train new models to classify the individual actions within the procedure (instead of the phases), so that we are able to explore the usefulness of this granularity level with respect to the assessment of declarative knowledge and advanced cognitive skills. 

Another limitation to our study was the lack of databases combining speech and video, which resulted in few videos available to test our models. To tackle this problem, we aim to create a database of speech and endoscopic video in surgical educational environments, which will likely be published to allow other researchers to work on this issue. This database will be used not only to adjust the weights of our models, if necessary, but also to test the validity of the proposed assessment system with surgical residents in the near future.

Furthermore, as mentioned in the introduction, we aim for this system to have training functionalities as well, such as alerting students in real time of missed steps and suggesting different pathways. Such a system may even be upgraded with a dictionary of possible solutions to complications encountered. 

Lastly, we could convert the desktop application to a web application, including a login system which allows for a database of different procedures performed by a student, such that not only they can have access to it, but will also allow for their teachers to easily track their progress.

## 4. Conclusions

In this study, we developed a pipeline to assess surgical procedural skills by means of a desktop application which uses a surgical phase classification algorithm to gather metrics from the procedure and present them visually to surgical trainers and trainees.

The feasibility of the algorithm and application were demonstrated as part of a preliminary analysis of the functionalities of the application. In the near future, we aim to test the validity of the proposed application as part of the assessment process of procedural skills (i.e., to find disruptions within the natural workflow of the procedure), as well as to test the feasibility to conduct real-time phase recognition to explore the potential of the application, and to provide residents with hints whenever disruptions from the normal workflow occur. 

We believe the application could have a major impact on the surgical educational community, since the human resources employed for the assessment of procedural skills will be reduced by much. In addition, the integrated SWA system could be used to provide formative feedback in real time and prevent mistakes in the overall procedure. Moreover, designers of procedural surgical simulators could benefit from the automatic workflows calculated in order to replicate different complications in the OR.

## Figures and Tables

**Table 1:** F1-scores obtained for the video to test the algorithm of combination of signals, of the video-based model and the speech-based model.

	F1-Score	Precision	Recall	Accuracy	Jaccard	Avg. Error/Phase	Avg. Error
Video-based model	0.775 ± 0.04	0.79 ± 0.042	0.785 ± 0.04	0.788 ± 0.089	0.682 ± 0.124	35.09%	37.24%
Speech-based model	0.785 ± 0.134	0.8 ± 0.099	0.78 ± 0.155	0.778 ± 0.157	0.68 ± 0.195	33.08%	23.71%
Complete algorithm	0.875 ± 0.049	0.875 ± 0.021	0.865 ± 0.021	0.819 ± 0.042	0.76 ± 0.028	11.86%	17.89%

**Table 2:** Comparison of methods and results of the main teams participating in the challenges trained using the same database we used in this study.

Year	Team	Basic Architecture	Temporal Component	Pretrained	F1-Score
**2021**	Digital Surgery	Encoder (ResNet50)-TCN Decoder	None	ResNet pretrained on CholecSeg8k	0.703
**2021**	2AI	Multi-task RCNN	None	Unknown	0.696
**2021**	UCL	Encoder (ResNet50)- MSTCN Decoder	None	ResNet50 pretrained with ImageNet, MSTCN pretrained on Cholec80	0.661
**2019**	HIKVision	ResNet50	LSTM	ResNet pretrained on ImageNet	0.6538
**2019**	CUHK	ResNet50	LSTM	ResNet pretrained on ImageNet, all pretrained with Cholec80	0.6498
**2021**	SIAT-CAMI	MCLNet	LSTM	Unknown	0.597
**2021**	Muroran-IT	ResNet18	None	Unknown	0.572
**2019**	MEVIS	ResNet50	LSTM	ResNet pretrained on ImageNet and Cholec80	0.543
**2021**	Wintegral	Resnet50	LSTM	Unknown	0.529
**2019**	NCT	ResNet50	LSTM	ResNet pretrained on ImageNet, all pretrained with Cholec80	0.49
**2019**	Wintegral	ResNet50	None	ResNet pretrained on ImageNet	0.4247
**2019**	CAMI-SIAT	Pseudo-3D Residual Network	None	Unknown	0.3865
**2019**	VIE-PKU	Parallelel ResNet10	None	ResNet and I3D pretrained on ImageNet, I3D pretrained on Kinetics	0.3329
**2019**	IGITech	ResNet50	None	Unknown	0.2393

## Data Availability

The speech data presented in this study are available on request from the corresponding author. The data are not publicly available due to privacy issues. The video data can be found in Synapse at https://doi.org/10.7303/syn18824884.

## References

[1] Verdura J., Maureen E., Ek S., Callery M.P. (2000). Systems, Methods, and Instruments for Minimally Invasive Surgery. U.S. Patent.

[2] Forde K.A. (1993). Endosurgical training methods: Is it surgical training that is out of control?. Surg. Endosc..

[3] Uemura M., Jannin P., Yamashita M., Tomikawa M., Akahoshi T., Obata S., Souzaki R., Ieiri S., Hashizume M. (2016). Procedural surgical skill assessment in laparoscopic training environments. Int. J. Comput. Assist. Radiol. Surg..

[4] Madani A., Vassiliou M.C., Watanabe Y., Al-Halabi B., Al-Rowais M.S., Deckelbaum D.L., Fried G.M., Feldman L.S. (2017). What Are the Principles That Guide Behaviors in the Operating Room?. Ann. Surg..

[5] Yule S., Flin R., Paterson-Brown S., Maran N. (2006). Non-technical skills for surgeons in the operating room: A review of the literature. Surgery.

[6] Yule S., Paterson-Brown S. (2012). Surgeons’ Non-technical Skills. Surg. Clin. N. Am..

[7] Jelovsek J.E., Kow N., Diwadkar G.B. (2013). Tools for the direct observation and assessment of psychomotor skills in medical trainees: A systematic review. Med. Educ..

[8] Vassiliou M.C., Feldman L.S., Andrew C.G., Bergman S., Leffondré K., Stanbridge D., Fried G.M. (2005). A global assessment tool for evaluation of intraoperative laparoscopic skills. Am. J. Surg..

[9] Neumuth T., Trantakis C., Eckhardt F., Dengl M., Meixensberger J., Burgert O. (2007). Supporting the analysis of intervention courses with surgical process models on the example of fourteen microsurgical lumbar discectomies. Int. J. Comput. Assist. Radiol. Surg..

[10] Lalys F., Jannin P. (2014). Surgical process modelling: A review. Int. J. Comput. Assist. Radiol. Surg..

[11] MacKenzie C.L., Ibbotson J.A., Cao C.G.L., Lomax A.J. (2001). Hierarchical decomposition of laparoscopic surgery: A human factors approach to investigating the operating room environment. Minim. Invasive Ther. Allied Technol..

[12] Loukas C., Gazis A., Kanakis M.A. (2020). Surgical performance analysis and classification based on video annotation of laparoscopic tasks. J. Soc. Laparoendosc. Surg..

[13] Gentric J.C., Trelhu B., Jannin P., Riffaud L., Ferré J.C., Gauvrit J.Y. (2013). Development of workflow task analysis during cerebral diagnostic angiographies: Time-based comparison of junior and senior tasks. J. Neuroradiol..

[14] Padoy N., Blum T., Feussner H., Berger M.O., Navab N. On-Line Recognition of Surgical Activity for Monitoring in the Operating room. Proceedings of the National Conference on Artificial Intelligence.

[15] Dergachyova O., Bouget D., Huaulmé A., Morandi X., Jannin P. (2016). Automatic data-driven real-time segmentation and recognition of surgical workflow. Int. J. Comput. Assist. Radiol. Surg..

[16] Twinanda A.P., Shehata S., Mutter D., Marescaux J., De Mathelin M., Padoy N. (2017). EndoNet: A Deep Architecture for Recognition Tasks on Laparoscopic Videos. IEEE Trans. Med. Imaging.

[17] Cadene R., Robert T., Thome N., Cord M. (2016). M2CAI workflow challenge: Convolutional neural network with time smoothing and hidden Markov model for video frames classification. arXiv.

[18] Jin Y., Dou Q., Chen H., Yu L., Qin J., Fu C.W., Heng P.A. (2018). SV-RCNet: Workflow recognition from surgical videos using recurrent convolutional network. IEEE Trans. Med. Imaging.

[19] Nakawala H., Bianchi R., Pescatori L.E., De Cobelli O., Ferrigno G., De Momi E. (2019). “Deep-Onto” network for surgical workflow and context recognition. Int. J. Comput. Assist. Radiol. Surg..

[20] Jin Y., Dou Q., Chen H., Yu L., Heng P.A. (2016). EndoRCN: Recurrent Convolutional Networks for Recognition of Surgical Workflow in Cholecystectomy Procedure Video.

[21] Lea C., Choi J.H., Reiter A., Hager G.D. Surgical Phase Recognition: From Instrumented ORs to Hospitals Around the World. Proceedings of the Workshop and Challenges on Modeling and Monitoring of Computer Assisted Interventions (M2CAI), Held in Conjunction with International Conference on Medical Image Computing and Computer Assisted Intervention (MICCAI).

[22] Cowan J. (2019). The potential of cognitive think-aloud protocols for educational action-research. Act. Learn. High. Educ..

[23] Suzuki T., Sakurai Y., Yoshimitsu K., Nambu K., Muragaki Y., Iseki H. Intraoperative Multichannel Audio-Visual Information Recording and Automatic Surgical Phase and Incident Detection. Proceedings of the 2010 Annual International Conference of the IEEE Engineering in Medicine and Biology Society, EMBC’10.

[24] Guzmán-García C., Gómez-Tome M., Sánchez-González P., Oropesa I., Gómez E.J. (2021). Speech-based surgical phase recognition for non-intrusive surgical skills’ assessment in educational contexts. Sensors.

[25] Guzmán-García C., Sánchez-González P., Oropesa I., Gómez E.J. Surgical Phase Recognition for Non-Intrusive Surgical Skills’ Assessment in Educational Contexts Based in Speech and Video. Proceedings of the XXXIX Congreso Anual de la Sociedad Española de Ingeniería Biomédica.

[26] Weede O., Dittrich F., Worn H., Feussner H., Jensen B., Knoll A., Wilhelm D., Kranzfelder M., Schneider A., Feussner H. Workflow Analysis and Surgical Phase Recognition in Minimally Invasive Surgery. Proceedings of the 2012 IEEE International Conference on Robotics and Biomimetics (ROBIO).

[27] Speidel S. EndoVis—Grand Challenge. http://www.endovis-grand-challenge.org.

[28] Zhang A. (2017). Speech Recognition (Version 3.8).

[29] Mikolov T., Chen K., Corrado G., Dean J. (2013). Efficient estimation of word representations in vector space. arXiv.

[30] Mikolov T., Sutskever I., Chen K., Corrado G., Dean J. Distributed representations ofwords and phrases and their compositionality. Proceedings of the 26th International Conference on Neural Information Processing Systems.

[31] Simonyan K., Zisserman A. Very Deep Convolutional Networks for Large-Scale Image Recognition. Proceedings of the 3rd International Conference on Learning Representations, ICLR 2015—Conference Track Proceedings.

[32] Vasershtein L.N. (1969). Markov processes over denumerable products of spaces describing large systems of automata. Probl. Inf. Transm..

[33] Wagner M., Müller-Stich B.-P., Kisilenko A., Tran D., Heger P., Mündermann L., Lubotsky D.M., Müller B., Davitashvili T., Capek M. (2021). Comparative Validation of Machine Learning Algorithms for Surgical Workflow and Skill Analysis with the HeiChole Benchmark. arXiv.

[34] Bodenstedt S., Speidel S., Wagner M., Chen J., Kisilenko A., Müller B.P., Maier-Hein L., Oliveira B., Hong S., Zamora-Anaya J. HeiChole Surgical Workflow Analysis and Full Scene Segmentation (HeiSurF). https://caruscloud.uniklinikum-dresden.de/index.php/s/z7jNWCHQ5TYfpSx.

